# Evolutionary principles and synthetic biology: avoiding a molecular tragedy of the commons with an engineered phage

**DOI:** 10.1186/1754-1611-6-13

**Published:** 2012-09-04

**Authors:** Eric G Gladstone, Ian J Molineux, James J Bull

**Affiliations:** 1 Section of Integrative Biology, The University of Texas, Austin, USA; 2 Section of Molecular Genetics and Microbiology, The University of Texas, Austin, USA; 3 Center for Computational Biology and Bioinformatics, The University of Texas, Austin, USA; 4 The Institute for Cellular and Molecular Biology, , Austin, USA

## Abstract

**Background:**

In prior work, adding a gene to phage T7 that degraded the host K1 capsule facilitated growth when plated on capsulated hosts. However, the transgenic protein (an endosialidase) is expressed as an exoenzyme, released from the cell at lysis but unattached to the phage particle. There is thus the possibility that the gene will be subject to a tragedy of the commons and be selected against, if the enzyme benefits other genomes.

**Results:**

This evolutionary perspective was supported in short term experiments. The genome carrying the endosialidase gene was favored on a capsulated host if grown in physical isolation of control genomes (lacking the gene) but was selected against otherwise.

**Conclusions:**

These results challenge efforts to engineer phages with exoenzymes that degrade biofilm polymers. If biofilms do not facilitate spatially structured phage growth, the transgenic enzymes may be rapidly eliminated from the phage population after release in the environment, even if the transgene benefits overall phage growth on the biofilm.

## Background

Among many exciting prospects for synthetic biology, one is to engineer microbes that degrade environmental compounds – pollutants, bacterial biofilms, or foodstuff for fuel. For many applications, it would be ideal if the engineered microbial population persisted and purveyed its beneficial role indefinitely, generation after generation. Yet, the successful engineering of a genome that performs our desired function is not sufficient to confer transgenic immortality. The engineering must be evolutionarily stable, so that natural selection does not favor loss of the novel functions. For some purposes, engineered microbes must also not be inferior to wild competitors, although competitive inferiority may be desired as a way of limiting the environmental impact of the strain. The competition factor is ecological, however, and is not relevant if the engineered microbes are released into a closed environment from which outside competitors are excluded.

The focus of this paper is the first problem: evolutionary stability. A sufficient condition to ensure evolutionary stability of an engineered modification would seem to be benefit to the organism, such as a drug resistance gene in the presence of antibiotics, because beneficial traits are maintained by selection. However, there is a particular class of benefits that are not necessarily evolutionarily stable: although the gene products are beneficial, the genes are not. This paradox arises from the subtle matter of *who* benefits from the gene product, whether it is specifically the individual carrying the gene or others in the population. If the beneficiaries include unrelated individuals as well as the bearer of the gene, a ‘tragedy of the commons’ may result in which the gene is selected against because the non-producers benefit without paying the cost of production [1,2]. In the parlance of evolutionary biology, the gene is producing a public good that benefits the whole.

These principles are broadly supported in the literature on evolutionary biology. It remains to be seen how relevant they are for engineering, and if they are relevant, how different engineering strategies may help solve them. We motivate our study with the specific example of bacteriophages engineered to degrade bacterial exopolymers. Lu and Collins [3] created a biofilm-degrading phage by endowing it with a gene for dispersin, an exoenzyme that degrades an essential biofilm component (*β*-1,6-*N*-acetyl-D-glucosamine). The inspiration for this engineering was that release of dispersin at lysis would provide high concentrations of enzyme where the enzyme was most useful for biofilm degradation, in the regions of highest cell density. Although the evolutionary advantage and evolutionary fate of the dispersin phage was not considered in that initial study, we might imagine that dispersin would be beneficial to the phage population by increasing the availability of hosts for the phage. However, even if dispersin provided such a benefit to the infecting phage population, the further question is whether the exoenzyme would be maintained over time or be lost in a tragedy of the commons. Here we test the basic principles behind this type of engineering. For practicality, we use an experimental system in which a phage produces an exoenzyme that degrades a capsular polysaccharide of its host [4].

### A system with conflicting individual and group benefits

**Figure 1:**
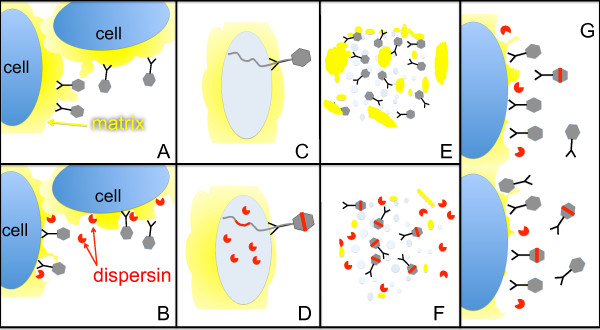
**Expected tragedy of the commons in an engineered phage system.** (**A**) Extracellular matrix (yellow) impedes phage attachment to the cell surface of its host. Phages are shown as stick figures with gray heads. (**B**) Exogenous endosialidase enzyme – from any source – augments phage infection by degrading the matrix and enabling phages to access the host surface. (**C**) The non-engineered (control) phage does not produce endosialidase. (**D**) The engineered phage produces endosialidase (endosialidase molecules in red; endosialidase phages with a red stripe). (**E**) Lysis of an infected cell by the control phage. (**F**) Lysis of an infected cell by the engineered phage releases free endosialidase molecules that degrade the local matrix and diffuse to other cells. (**G**) The tragedy of the commons. In a mixed environment, endosialidase benefits both types of phages equally, and the control phage numbers should evolve to exceed engineered phage numbers over time because only the engineered phages experience the reproductive cost of producing the enzyme.

A phage’s production of a compound that facilitates infection of new hosts will benefit that phage, provided the enhanced access to hosts is not outweighed by the cost of production. If the compound is released as a free molecule into the environment (as applies here, Figure 1), then the compound will benefit all phage in the local group, not just the producers, because all phage have increased access to hosts. Yet within the group, the non-producers have the reproductive advantage because they avoid the metabolic cost of producing the compound. Over time, non-producers will continually outgrow the producers in the group even as the benefit to the group collapses: all phages share equally in whatever group benefit remains, but only the non-producers have the individual benefit of avoiding production cost.

This type of system should obey a tragedy of the commons [5], a process in which unchecked selfish interests of the individual lead to overexploitation and eventual collapse of a common resource that benefits the entire group. In Hardin’s original paper, the model was illustrated mostly with examples in which the public good was a pre-existing natural resource, but the paper also extended the process to public resources created by society (e.g., parking spaces, banks) as well as to evolutionary processes that favor the individual over the group. The metaphor has been so widely adopted in evolutionary biology that it has become virtually synonymous with a conflict between group and individual fitness, and indeed, our study can be viewed in this latter context without invoking a tragedy of the commons. In our study, the common resource benefitting the group is a phage-produced diffusible enzyme that degrades host capsule, allowing rapid infection of new hosts by any phage in the local group. The selfish behavior is the failure of an individual phage genome to produce enzyme, with a direct analogy to humans failing to support a public good by not paying taxes.

A simple formulation augments these arguments. The intent of the model is to foster intuition rather than to yield a formal description of the phage system. The nature of selection on the transgenic phage can be reduced to two effects. For an individual lacking the transgene, fitness over one generation is the product *fh*, where *f * is fecundity, the number of progeny produced from a single infection, and *h* is the access to hosts, a per progeny probability of infecting a host (*h *< 1). We let fitness of the transgenic type be *FH*. Compared to the genome lacking the transgene, a transgenic infection has lower fecundity (*F* <*f*) due to the cost of producing the enzyme, but has a higher probability of infecting a host because the enzyme increases access (*H* >*h*). We can thus think of *H*/*h* as the benefit (*B*) of the transgene and *F*/*f* as its cost (*C*), because *H*/*h* should be greater than 1 and *F*/*f* should be less than 1. The transgenic phage has the higher fitness if

(1) FH>fh,

hence if

(2) BC>1.

In a real system, these quantities will not be static. For phage growth in a confined environment such as a plaque or a small volume of liquid, the benefit *B* is likely to change with time since infection/inoculation, as phage density increases and protein concentrations build. If *B* varies with time, the overall growth of the transgenic phage depends on the average *B* over the interval. Thus the net effect of selection may depend on how long the competition lasts.

**Figure 2:**
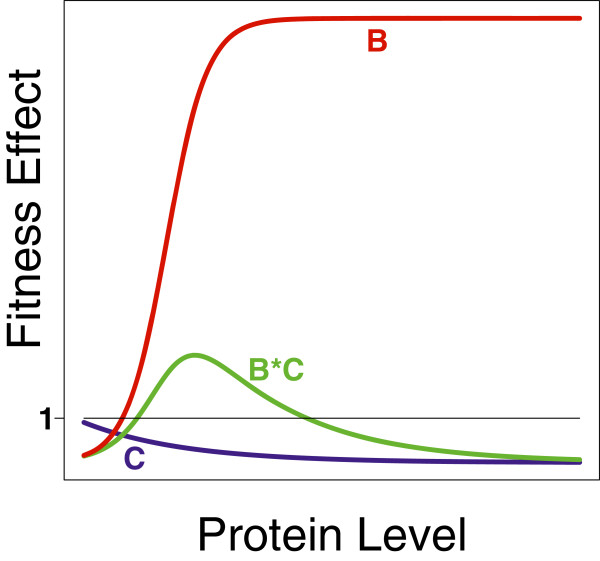
**Net fitness effect of a transgene.** Hypothetical relationship between cost function (blue, curve labeled C), benefit function (red, B) and their product (green, B*C). If B is saturating but C is not, then the transgenic phage will outgrow the unmodified phage only at low-intermediate levels of gene expression.

*B* and *C* may also be environment specific and will certainly depend on gene expression level (a hypothetical case is illustrated in Figure 2). With regard to gene expression level, benefits may often be saturating with product concentration, whereas the burden of expression (cost *C*) should increase with expression level, perhaps more than linearly. Consequently, the net fitness of a transgene may be highly sensitive to various conditions, especially if the benefit is quantitative rather than absolute. Importantly, implementations based on our intuition about suitable genes for beneficial transgenesis may often fail due to these quantitative issues rather than to an intrinsic misunderstanding of the qualitative effects.

The model has thus far implicitly compared fitnesses of separate populations. Evolutionary inferences based on these comparisons thus assume that evolution is from competition between separate populations (groups) of each phage type, as when the two are grown in isolation. If the enzyme of the transgenic phage is produced as a free, diffusible molecule, that enzyme benefits not only growth of its own genome but also benefits the growth of any other (non-transgenic) phages in the local group – the enzyme pool is effectively a commons that can benefit all. In a population of fully mixed individuals of both types, the non-transgenic phage will always have the higher fitness because both types benefit equally from the enzyme – the group benefit – yet only the transgenic phage pays the cost of production. That is, both phages will experience *H* host encounters, but the difference in fecundity, *F* versus *f *, is unaffected. In a group with both types, *fH* applies to the non-transgenic phage and always exceeds *FH* for the transgenic phage. This asymmetry drives the tragedy of the commons. (The value of *H* in a mixed group would of course depend on the abundance of the transgenic phage, so a more formal argument would specify *H* as a function of phage density; the important point is that, whatever the value of *H*, it is shared between the two phage types.)

From these simple considerations, there are two ways to avoid a tragedy of the commons and maintain the transgenic individuals in the long term. One is to grow them in a spatially structured environment, so that the benefit of the enzyme (*H*) accrues only to those genotypes producing the enzyme - that even though the enzyme is produced as a free molecule, it cannot diffuse far enough to benefit other types. This strategy is one of group selection and works in the long term only if the populations are repeatedly dispersed into small, isolated subpopulations so that any enzyme-lacking mutants of the transgenic phage have little time to ascend before being required to grow on their own [6,7]. The other solution is to abolish the potential for a commons, as by engineering the enzyme so that it is not a free molecule but is chemically coupled to the producing genome; then *H* remains associated with the transgene even in a mixed population. The purpose of the experimental design is to see if these principles can be successfully applied in a transgenic phage system.

## Results

### Setup and general observations

Most experiments were done with two phages, T7E_1_ (carrying endosialidase) and T7_0_ as a control. T7_0_ carried a short insert in frame (an S-tag) considered to be of little consequence, whereas the engineered phage, T7E_1_, was cloned with the tailspike gene from phage K1-5 that encodes an endosialidase. The only strict requirements for the control phage are that its intrinsic growth be superior to that of T7E_1_ and that it benefit from the presence of exogenous endosialidase. It would have been feasible to use a wild-type T7 as the control, but use of the same genomic backbone for both phages ensured that fitness differences could be attributed largely to the engineered differences; the wild-type phage appears to have a substantially higher fitness than the vector (data not shown), an effect that would have complicated finding a suitable dynamic range for competing a wild-type control with an engineered vector.

**Figure 3:**
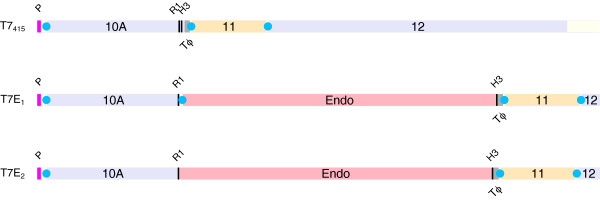
**Phage design.** The part of the T7 genome subjected to cloning is shown, drawn to scale (approximately 4.5kb shown of a 40kb genome). (T7_415_) The cloning vector, indicating the locations of the promoter (pink) in front of gene 10A, all ribosome binding sequences (RBS, blue dots), and the 3 phage genes in the vicinity of the cloning region. The T7 terminator T*ϕ*is shown in gray, and the two restriction sites used for cloning are indicated with black, vertical lines (R1 is for Eco RI, H3 is for *Hind* III). (T7E_1_) The endosialidase was cloned between the Eco RI and *Hind* III sites with its own RBS and a stop codon terminating translation of 10A. The T7 terminator was located downstream of the cloning. (T7E_2_) The endosialidase was cloned between the Eco RI and *Hind* III sites as an inframe fusion with 10A. but the only clones obtained contained an evolved stop near the end of *10A*. The T7 terminator was shifted downstream of the insert. Stops are present at ends of all genes in all 3 genomes (*10A*, Endo, *11*, and 12). The control phage T7_0_ is the same as T7_415_ with a 15 amino acid S-tag extension between the Eco RI and *Hind* III sites (flanked by single amino acids).

Transcripts in T7 extend across many genes [8]. The endosialidase in T7E_1_ was encoded as a separate protein behind gene *10A*. The design included a stop at the end of *10A*, followed by the native ribosome binding sequence (RBS) of the endosialidase, then followed by the endosialidase gene itself. The nearest upstream promoter was the native T7 promoter in front of gene *10* (Figure 3). Thus all transcripts encoding endosialidase would have encoded gene *10A* in front. Relative protein expression levels of gp10 and endosialidase would then have depended on relative strengths of the RBS sequences; computational analysis suggests that the RBS strengths are nearly equivalent, within a factor of 1.3 (using the method of [9]; see also [10]).

The selective host was EV36, an *E. coli* K12 strain intrinsically permissive for T7 but expressing a K1 (sialic acid) capsule [11]. The capsule acts as a barrier between the phage and its bacterial receptors. Expression of the capsule thus protects the cell from the phage, and degradation of the capsule by the phage-encoded endosialidase augments infection. However, although the endosialidase protein made by T7E_1_ is the tailspike of phage K1-5 (see Methods), it does not attach to the T7 virion and thus is released as free protein. Consequently, the endosialidase produced by an individual T7E_1_ specifically helps that genome’s progeny infect new EV36 only to the extent that the diffusing phage progeny remain spatially associated with the sialidase molecules after release at lysis.

Furthermore, there is a potential ‘chicken and egg’ problem with this phage. Given a suspension of phage particles, some host cells must be susceptible to T7 in the absence of enzyme so that the first generation of infections will occur to produce enzyme. If the capsule cannot be breached without enzyme, then phages alone cannot get the process started [4]. The success of T7E_1_ in our system thus depends on the presence of some cells that can be infected before capsule degradation.

Scholl [4] reported that T7 lacking the sialidase gene could not form plaques on EV36 grown in LB broth, whereas T7 with the sialidase gene formed plaques occasionally, at an efficiency of 10^−3^ to 10^−4^. In our hands, both T7E_1_ and T7_0_ could form nearly normal plaques on EV36 grown in LB. With some effort, we found growth and plating conditions for EV36 that greatly impaired plaque formation by T7_0_ (M9 glucose). A performance difference between alternative sources of LB media is not necessarily surprising, because LB is not a defined media and ingredients from different manufacturers are now known to vary in sRNAs that affect bacterial gene expression [12].

### The transgenic phage T7E_1_ is at a disadvantage on a non-capsulated host

**Figure 4:**
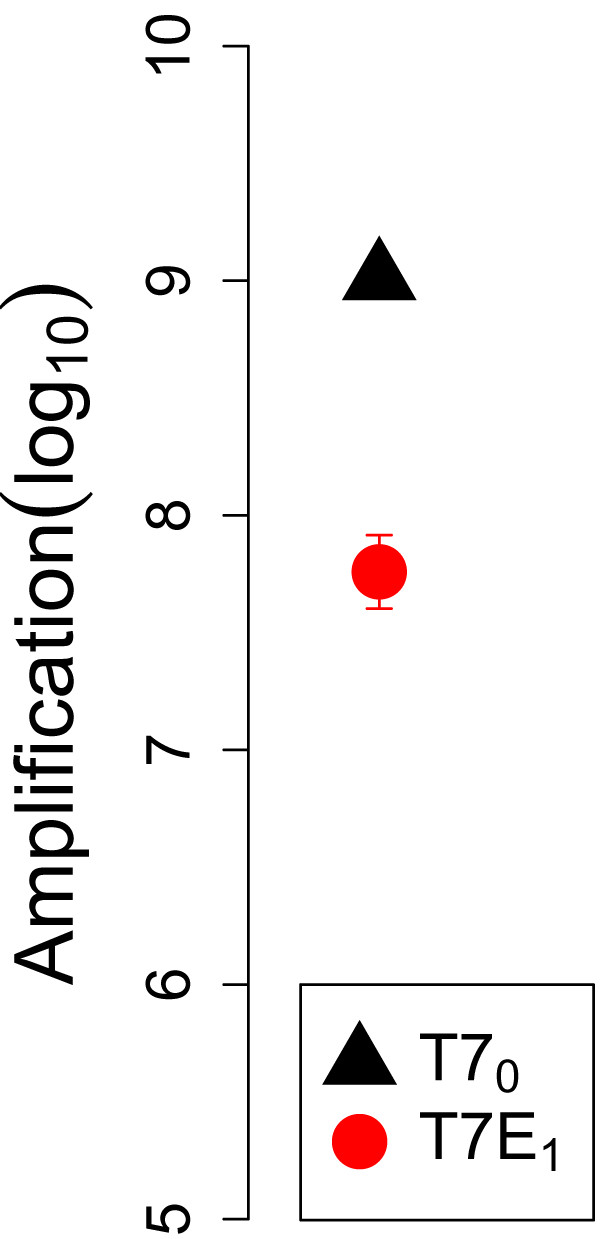
**Amplification on non-selective hosts.** Amplification of T7_0_ is 20-30 fold superior to that of T7E_1_ on a host lacking the K1 capsule (P < 0.001, t(6) = 7.4). The inferior amplification of T7E_1_ is attributed to a cost of encoding the endosialidase protein when there is no benefit of the protein. The growth period here spans ≈ 4 phage generations, and the media was LB agar with the non-capsulated host IJ1133. The bars span 2 standard errors, although the bars on T7_0_ are small enough to be obscured by the triangle.

A tragedy of the commons stems from selfish behavior of some or all individuals in the group. In an evolutionary context, the selfish behavior needs to benefit the selfish individual, or it cannot possibly spread. In our study, the selfish genome is the one lacking the endosialidase (the control phage, T7_0_). Thus, for this system to provide a legitimate test of the tragedy of the commons model, the control phage must have higher intrinsic fitness than T7E_1_. A higher intrinsic fitness means a higher growth rate when endosialidase is not needed or is available from another source. This condition can be tested by competing T7E_1_ and T7_0_ on a host permissive to T7 that lacks the capsule - where there is no possible benefit of the endosialidase for access to hosts. Competitions done ‘separately’ on IJ1133 support this assumption, with a 20-30 fold greater amplification by T7_0_ (Figure 4; separate competitions ensure that the enzyme produced by T7E_1_ cannot affect growth of T7_0_, so a superiority of T7_0_ must mean that enzyme production constitutes a net detriment to T7E_1_ under these conditions). Comparisons reported below also support an intrinsic superiority of T7_0_.

### Separate competition on the capsulated host favors the endosialidase phage T7E_1_

**Figure 5:**
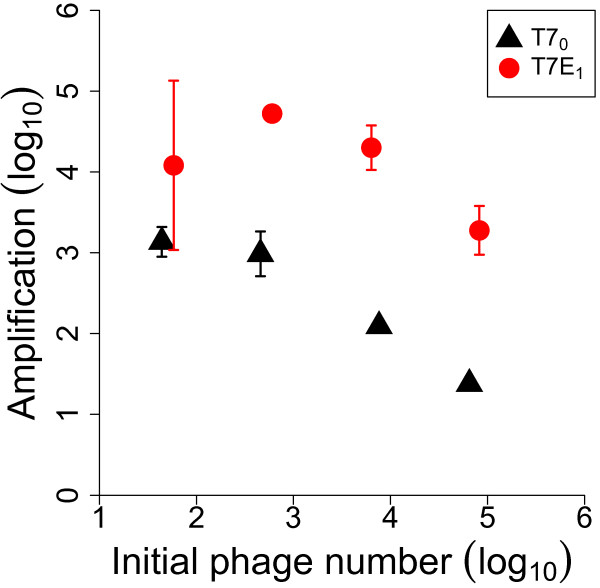
**Separate amplification on selective hosts.** Amplifications of T7_0_ and T7E_1_ on the capsulated host (EV36 in M9) when grown in physical isolation of each other. There is a superiority of T7E_1_ at all initial phage densities tested, although the difference is (highly) statistically significant at only the 3 highest densities in individual, 2-tailed t-tests (tests were performed on differences between each pair of T7_0_ and T7E_1_ samples most similar in initial density). This assay demonstrates that the endosialidase protein enhances phage growth on the capsulated host. Amplifications here are lower than in Figure 4 because the host and media used here (M9 glucose) are not as conducive to cell growth. Assays were paired, so that T7E_1_ and T7_0_ amplification samples were taken from different zones on the same plates. Bars span 2 standard errors.

The preceding result established that T7E_1_ amplifies less than T7_0_ on hosts lacking a capsule. If T7E_1_ is ever to outgrow T7_0_, it must be in circumstances when the endosialidase is specifically beneficial, as when capsule is a significant barrier to infection. For this assay, we calculated phage amplifications on the capsulated host (EV36 grown in M9 glucose) for different initial concentrations of phage. The phage suspension was either of pure T7E_1_ or pure T7_0_. These separate competitions supported a superiority of T7E_1_ over T7_0_ across a range of initial phage densities on the capsulated host (Figure 5).

### The tragedy: mixed competition selects against the sialidase phage T7E_1_

A tragedy of the commons was avoided in the preceding experiment because the T7E_1_ phage type was grown separately and grown so briefly that mutants lacking the endosialidase could not arise and take over. As the endosialidase is produced as a free molecule, the system should be prone to a tragedy of commons when the two phages are grown together: T7_0_ should outgrow T7E_1_ on a capsulated host when the two phages are mixed, because (1) T7_0_ has an intrinsic growth advantage and (2) it can use the endosialidase produced by T7E_1_ when both phages are mixed. This prediction assumes that there is free mixing of endosialidase in the local environment infected by multiple phage types.

**Figure 6:**
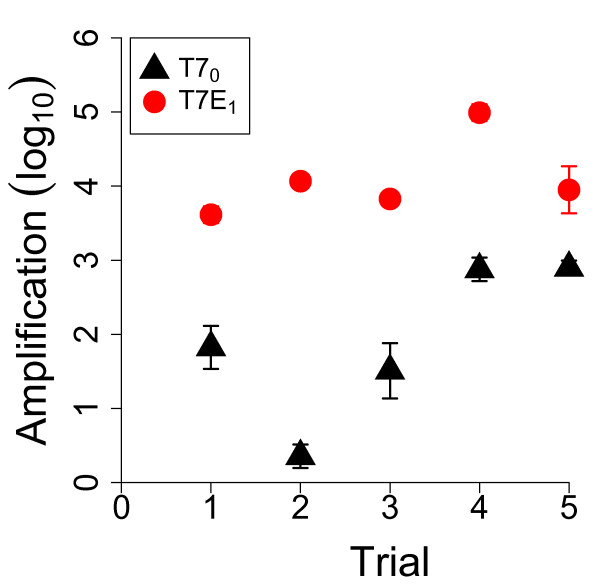
**Amplification on selective hosts comparing separate and mixed competitions.** (**6A**) Comparison of T7E_1_ and T7_0_ amplifications when grown in separate competitions. Five independent amplification measurements for the two phages are given; initial titers ranged 2−8×10^4^. These data are independent of those in Figure 5; the initial phage titers here fall between the rightmost two X-axis values in that figure. All 5 assays exhibit a (highly) statistically significantly greater amplification of T7E_1_ than of T7_0_. Bars span 2 standard errors. (**6B**) Changes in relative frequencies when grown in mixed competitions: a tragedy of the commons. The figure shows the proportions of T7E_1_ before and after growth for 5 trials, the arrows indicating that the relative proportion of T7E_1_ declined in all cases. The relative decline is interpreted as stemming from T7E_1_ producing an enzyme that benefits itself and T7_0_ equally but only T7E_1_ experiencing the cost of providing the benefit. In all 5 cases, the proportional increase of T7_0_ is statistically significant by a Fisher^*′*^s exact test or *χ*^2^test; all are highly significant except the second, which is marginally significant. From left to right, each mixed assay trial in (**6B**) corresponds to the respective trial in (**6A**), done in different zones on the same plate. All assays used EV36 with M9 glucose media.

Mixed competitions were done in parallel with separate competitions, all at approximately the same density of initial phage (2×10^4^−8×10^4^) and on the capsulated host (EV36 grown in M9 glucose). As also in Figure 5, the transgenic phage T7E_1_ exhibited significantly higher amplification than the control phage T7_0_ in separate competitions (Figure 6A); yet the transgenic phage fared worse than the control phage in mixed competitions (Figure 6B). By using the same protocol for mixed and separate competitions, other than phage mixing, a difference in the relative fate of T7E_1_ should be uniquely attributable to the presence or absence of the control phage, T7_0_. For each of the 5 replicates, a separate and mixed assay were performed in parallel on the same plate, to reduce unwanted sources of variance.

### Alternative engineering: phage T7E_2_

From Figure 2, the level of gene expression may determine whether a gene capable of providing a benefit actually offers a benefit to the recipient genome. Although T7E_1_ was engineered to carry a gene beneficial for growth on a capsulated host, its level of expression was not optimized - the level of expression would have been high, similar to that of the major capsid protein, the gene immediately upstream (see above). Furthermore, a different method of cloning might be employed to avoid a tragedy of the commons altogether – as in coupling the endosialidase to the virion. It is thus expected that alternative designs for a transgenic phage could potentially yield better growth on the capsulated host.

A second transgenic design was implemented, designated T7E_2_, in which the endosialidase was cloned as a C-terminal fusion to the major capsid protein (Figure 3). If the cloning worked as designed, T7E_2_ would create phage heads in which all major capsid proteins were fusions with endosialidase. The isolates recovered all carried a base substitution that effected a stop codon at the end of *10A*, terminating translations without any endosialidase residues. Although we might have hoped otherwise, this outcome is unsurprising because the 811 residue endosialidase protein is so large as to interfere with assembly if most capsid proteins carry it (the major capsid protein is 344 amino acids long). Although the vast majority of capsid proteins in the isolates of T7E_2_ recovered would have lacked residues of sialidase, fusions of capsid and sialidase would have resulted from low-level translational read through of the stop (maybe 1%, e.g., [13]), as a C-terminal domain on a small fraction of major capsid proteins. As pointed out by a reviewer, cryptic RBS activity immediately upstream of the endosialidase start (within the protein coding sequence of 10A) could also have allowed expression of endosialidase, and in this case, it would not have been fused to the capsid protein. Computational analysis of translational initiation of the 30 bases preceding the endosialidase start indicated a 15-fold lower rate than of the gene *10* rate, so a low level of unfused endosialidase expression is indeed a possibility. T7E_2_ exhibited endosialidase activity in phenotypic assays (data not shown), but we did not assess whether the protein with this activity was of the expected size for endosialidase versus endosialidase fused to capsid protein.

There are thus two possible differences between T7E_2_ and T7E_1_: the level of endosialidase production is perhaps two logs lower in T7E_2_, and the endosialidase may be fused with the capsid protein. As a consequence of the latter property, a third possible difference is that some of the endosialidase molecules might be attached to the phage head rather than be released as free proteins (albeit fused with capsid proteins). We did not determine whether any capsid-sialidase fusions were incorporated into the phage head.

**Figure 7:**
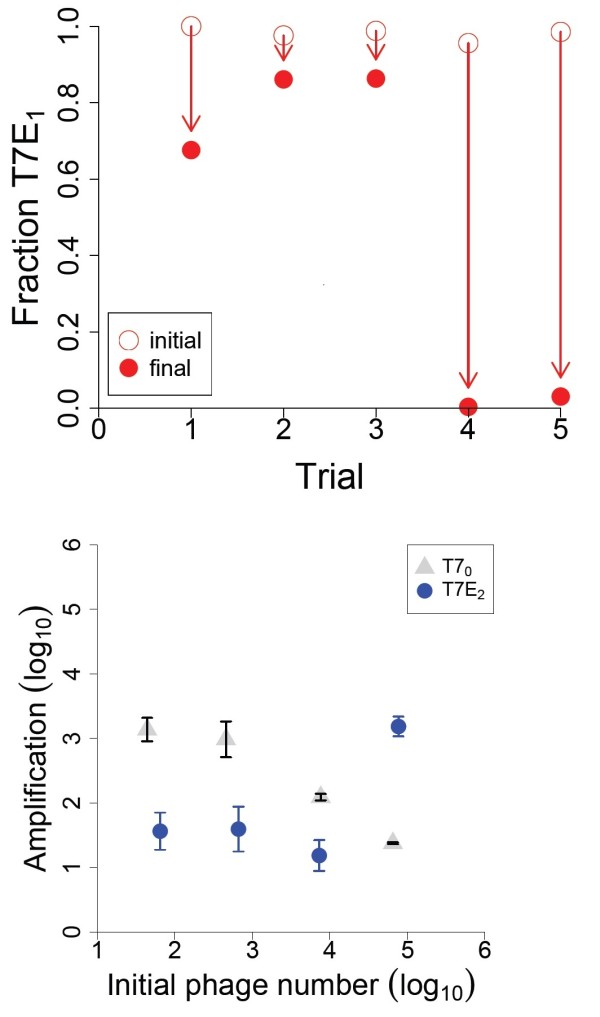
**Amplification with alternative engineering.** Amplification on the capsulated host of T7E_2_ (blue circles), for comparison to T7_0_ (black diamonds). T7E_2_ shows lower amplification except at the highest initial concentration. Paired assays of T7_0_ and T7E_2_ were done on the same plates, so the difference is not attributable to plate-plate variation. Data for T7_0_ are the same as in Figure 5. Host was EV36 with M9 glucose media. Tests were performed between the pairs of T7_0_ and T7E_2_ samples most similar in initial density; all differences are statistically significant. Bars span two standard errors.

A separate competition was done between T7_0_ and T7E_2_ (using EV36 grown in M9 glucose). Except at the highest initial concentration of phage (≈10^5^), T7E_2_ amplified less than T7_0_ (Figure 7). This fusion cloning strategy is thus substantially inferior to that of T7E_1_, except at the highest initial density. The data also suggest a substantial cost to endosialidase production in T7E_2_, because that phage amplifies almost 30-fold less than does T7_0_ at the lower initial densities. A low level of endosialidase production is unlikely to be metabolically expensive, so there may be some interference of phage assembly caused by the capsid-endosialidase protein. Indeed, this observation provides indirect support for the presence of endosialidase as a fusion with capsid.

## Discussion

The fate of a beneficial gene cloned into a bacteriophage genome obeyed evolutionary principles considered to underlie the natural selection of a group benefit. The transgene encoded an enzyme, an endosialidase, that degrades a sialic acid (K1) capsule found on some host strains. The protein thus improved phage infection rates. If the gene was cloned as a free protein, released into the environment at lysis, the engineered genome (T7E_1_) outgrew the control phage (T7_0_, lacking the endosialidase) when two conditions were met: (1) The host expressed the K1 capsule. (2) The engineered phage was grown in physical isolation from the control phage. Mixing the two phages before growth or using a host lacking the capsule reversed the outcome. Our cloning the enzyme as a presumed fusion with the phage capsid protein resulted in superior growth over the control under these same conditions but only in a narrow range of high initial phage densities. However, it is also possible that the active enzyme in this latter case was in fact an endosialidase not fused with capsid, due to a low level of cryptic translation initiation, or that the fusion protein was not included in the capsid.

The ability of high concentrations of free endosialidase to strip K1 capsules and augment phage infection has precedent in previous work. Artificial infections of mice with K1 capsulated bacteria can be cured with high doses of exogenous endosialidase; this effect was interpreted as enzyme-mediated capsule degradation enhancing immune system clearance of the bacteria [14]. Also, in vitro growth of a wild siphovirid phage (not related to T7) on a K1 capsulated host was enhanced by addition of free endosialidase to the media [15]. These prior observations led to our design and led us to anticipate the outcome here. Nonetheless, a different outcome might well have been obtained here from the effects of several unmeasured variables (e.g., [7,16]), and indeed, different ecological methods of creating spatial structure can lead to qualitatively different outcomes [6,17]. Furthermore, there are ecological effects of depolymerase enzymes that can affect phage dynamics [18]. The main question both addressed and answered here is that a superficial understanding of basic evolutionary and engineering principles was robust enough to predict a qualitative outcome.

The results support theory and experimental work underlying a phenomenon generally known as a tragedy of the commons, which is more broadly interpreted as the evolution of cooperation or of group benefit. In a tragedy of the commons, individual selfish interests conflict with group benefit and prevent the evolution of traits that benefit the group. Experimental evidence supporting different aspects of the theory has been provided in the context of bacterial siderophores, experimental phage and plasmid evolution [1,2,6,19,20] and indeed is commonly observed as the presence of ‘satellite’ colonies when selecting ampicillin resistant plasmids in a transformation library. The biological context here differs in details from these precedents, but ours is also the first demonstration that the principles apply to the fate of a cloned gene outside of its natural context. Cloned genes may behave differently than native genes because cloned genes have not evolved to be integrated into the network of genomic interactions and thus may have fewer pleiotropic effects than native genes.

The experiments here have explored only a few of the many possible environmental sensitivities and quantitative difficulties in maintaining a group-beneficial transgene. The net benefit of a gene will generally depend on its level and timing of expression, the duration of the growth phase during which selection is operating, and on changes in the external environment that affect the momentary benefit [16]. In the present context, we attempted several variations in growth media before finding one that consistently allowed the engineered phage to outgrow the control phage. It is possible that this sensitivity in outcome reflected the thickness or other properties of the bacterial capsule that varied with growth conditions, but the situation may be considerably more complicated than suggested by our limited knowledge. Furthermore, two alternative methods of cloning the same gene led to quantitatively different evolutionary outcomes, which might have been due to how the cloning affected protein levels or to different molecular contexts for the protein (a free endosialidase versus an endosialidase fused to the capsid protein).

The second cloning method used here was motivated by one solution to avoid a tragedy of the commons: physically couple the beneficial gene product with the genome producing it. Attaching the endosialidase to the phage particle ensures that the engineered genome benefits from its transgene, although it is not clear if our attempted implementation of this method (endosialidase fused to capsid protein) indeed achieved this objective. Even so, coupling the transgenic protein to the viral particle does not ensure that the benefit goes entirely to the engineered genome: phage assembly is relatively inefficient, and lysis releases many unassembled components along with complete virions. Likewise, co-infection of a cell by multiple phages would allow one phage to capture the enzyme from another. Thus no cloning strategy is completely immune to a tragedy, but some are less prone than others.

The understanding achieved here may facilitate designing phages for controlling biofilms. One such original effort was the cloning of a dispersin gene into T7 [3]. Dispersin degrades an essential component of *E. coli* biofilms, so the engineered phage ought to be able to penetrate biofilms and deliver dispersin to successively deeper levels, ultimately destroying the biofilm. Short term assays of 24hr biofilms suggested that the transgene enhanced biofilm destruction. The question raised by the present study is whether the dispersin transgene would persist in the long term growth of phage in a biofilm. Produced as a ‘public good,’ the dispersin gene’s fate would depend on phage dynamics and spatial structure of phage growth in the biofilm, which is presently unknown and may even vary from biofilm to biofilm; in particular, phage attacking a biofilm may persist by infecting the planktonic cells released from the biofilm rather than by penetrating it [21]. Whereas the spatial structure of a biofilm-infecting phage is unknown, we can invert the model and argue that long term fate of the dispersin-encoded transgenic phage in a biofilm can reveal the nature of phage population structure. However, spatial structure is a necessary condition for dispersin maintenance, not a sufficient one: failure of a transgenic dispersin to be maintained in T7 grown on a biofilm could stem from many causes even with spatial structure.

Even when a transgene is initially beneficial to its recipient genome, the engineering is unlikely to be without flaws. Directed evolution may then be employed to improve the engineered genome. For example, growth of our endosialidase phages under selective conditions might lead to improvements that broaden the spectrum of environmental conditions maintaining the transgene. Yet perpetual growth of a transgenic organism may favor improvements of some types but not others, and the tragedy of the commons perspective offers insight to which types of improvements may be expected. For example, mutations improving transgene expression, where the benefit of improved expression is manifest within the cell as increased burst or decreased lysis time, should not be subject to a tragedy of the commons, because any benefit is realized specifically by the mutant genome.

In contrast, mutations improving the extracellular performance of public good proteins (such as endosialidase activity) may suffer a tragedy of the commons even with spatially structured growth. A mutation creating a more efficient endosialidase would usually arise in a swarm of non-mutant enzymes produced by neighboring relatives such that its benefit is shared across a large population of phages. Only if the mutant phage was grown in isolation from others would it be able to benefit from its superior mutation. Thus, the tragedy of the commons guides both the maintenance of the engineered change as well as its improvement.

A tragedy of the commons does not preclude use of engineered genomes for the intended purpose. The rate of evolution away from the engineered state will dictate how long an infusion of transgenic organisms has its intended effect. As noted by a reviewer, decay of the engineered state can be temporarily reversed by re-introduction of engineered genomes. Where spread of the engineered gene to a natural fauna is to be avoided, an engineered senescence of the transgene may even be desirable.

## Conclusions

The short term evolutionary fate of a gene cloned into a phage genome and expressed as a free protein obeyed principles underlying the evolution of cooperation. The gene encoded an endosialidase that degraded host capsular polysaccharides (K1) and was expected to expedite phage initiation of infection. The gene was highly disadvantageous when grown on a host lacking the capsule, but it was also disadvantageous on a capsulated host when the engineered phage was grown in a mix with the control phage lacking the endosialidase gene. The latter effect is a molecular tragedy of the commons, in which the free enzyme – the gene product – benefits all genomes in the local environment, even those that do not carry the gene and do not pay the cost of producing the enzyme.

There are two solutions to this tragedy of the commons. One is to employ ‘group selection,’ growing each phage type in isolation of the other. For phages released into the environment, the ecology will be beyond the control of the engineer and may not allow this solution. The second solution is to clone the transgene so that it is not expressed as a free protein, but is physically coupled to the genome producing it. Only the former approach was broadly successful here, but our attempted implementation of the second approach likely created problems with viral assembly and, even then, did not obviously achieve the objective of endosialidase coupled with the phage particle.

The evolutionary principles germane to this study apply broadly. Thus, a tragedy of the commons is possible with other enzymes cloned into phage genomes and even with secreted molecules of unicellular organisms. Efforts to engineer microbes with the capacity to release beneficial molecules into their local environment may thus need to account for – or modify – the organism’s ecology, or find ways of tethering the extruded molecules to the producer.

## Methods

### Strains

Two bacterial strains were used (Table 1). IJ1133 [22] is a capsule-free *E. coli* K12 strain permissive for T7 and has been used in many other studies of ours [23]. EV36 is an *E. coli* K12 strain that is permissive for T7 but encodes a K1 capsule (polysialic acid) that can retard or block infection by T7 [4,11]. Prior to this study it was unknown how growth characteristics (e.g., media) of EV36 affect infection dynamics of T7.

**Table 1:** Strains

**Designation**	**Characteristics**	**Reference**
EV36	*E. coli* K12 with K1 capsule	[11]
IJ1133	*E. coli* K12 fully permissive to T7 and lacking the K1 capsule	[22]
T7_0_	T7 Select 415 with 15 AA control insert and C74 left end	
T7E_1_	T7 Select 415 with sialidase insert expressed as free protein, C74 left end	cf. [4]
T7E_2_	T7 Select 415 with sialidase insert expressed as stop codon readthrough fusion to *10A*, C74 left end	

Three phages were engineered (Table 1). All used the T7 Select 415 vector (Invitrogen) as a backbone [10]. The T7 capsid contains 415 copies of the capsid protein, and the ‘415’ designation refers to the fact that all capsid proteins in the virion are derived from this vector. This phage is deleted for 2600 bases of the wild-type genome, mostly in the phage early region (‘left’ end), but our vector left end was exchanged with that of a phage carrying the C74 deletion [24] by a *Bcl* I fragment swap; this fragment carries an intact *0.3* gene, which enables the phage to grow on Type I RM hosts (which we used for work not reported here). The vector also includes a cloning site at the 3’ end of the major capsid gene *10A*, deleting the unique, non-essential portion of the minor capsid gene *10B*.

Our control phage, T7_0_, was modified to carry the short Invitrogen 15 amino acid control sequence in the cloning site between the *Eco* RI and *Hind* III sites, flanked by single amino acids; we consider this sequence to be of little consequence, and indeed, its nature is important only in that it does not degrade the K1 capsule. Phage T7E_1_ was modified to carry the full endosialidase gene and its ribosome binding site of phage K1-5 [25] between the *Eco* RI and *Hind* III sites of the vector; the fragment was amplified by PCR from phage K1-5 to have *Eco* RI and *Hind* III ends and to insert a stop codon in frame with *10A* in addition to 30 bases of K1-5 sequence upstream of the endosialidase start. Phage T7E_2_ also carries the K1-5 endosialidase gene between *Eco* RI and *Hind* III sites, but was engineered so that its initial Met codon was in frame with *10A* as the first unconstrained codon beyond the *Eco* RI site. The difference in these latter two designs is that T7E_1_ encodes sialidase as a free protein, released at lysis; T7E_2_ is expected to encode endosialidase as a protein fusion with *10A*, but the only clones obtained had acquired a stop codon immediately in front of the endosialidase reading frame. The nature of the cloned endosialidase gene was verified by Sanger sequencing.

### Media

LB broth (10 g NaCl, 10 g Bacto tryptone, 5 g Bacto yeast extract per L) and M9 glucose (47.8 mM Na_2_HPO_4_, 22 mM KH_2_PO_4_, 8.5 mM NaCl, 1.87 mM NH_4_Cl, 1 mM MgS0_4_, 0.1 mM CaCl_2_ with 0.2% glucose) were used. Plates used the indicated media with hard agar at 15g/L Bacto Agar, soft agar at 7 g/L Bacto Agar.

### Phage competitions

Phage were competed in growth assays on plates. Assays on host EV36 were done on M9 glucose plates. EV36 was grown 24hr to high density (at least 10^9^/mL) in liquid M9 glucose at 37°C. Fifty microliters of the suspension were added to 3 mL soft agar and poured on hard agar. After the top agar gelled, a small volume of phage suspension (2-8 *μ*L) was applied to the surface at a defined point, and the pipette tip depressed into the soft agar to enhance diffusion. Plates were incubated 16hr at 37°C. After growth, the zone of top agar surrounding the site of phage application was scraped into a known volume of media (with chloroform to kill cells), vortexed, allowed to sit 1hr, and plated to determine titer. Two types of assays were done in this fashion with M9 glucose media, differing only in whether the initial phage suspension was of a pure phage type (‘separate’ competitions) or a mix of phage types (‘mixed’ competitions).

Separate competition assays were also done on IJ1133 in LB. In these assays, phage were plated at low density and grown at 37°C for 24hr. Plaques were counted to determine initial titer, and the top agar was scraped into media (plus chloroform) to determine total phage numbers.

The growth rate of a phage in the separate competition treatment was measured as the number of phage recovered from the infected zone of a plate divided by the number of phage initially added to that zone - the total amplification. In mixed competitions, the proportion of each phage type was measured in the pool of phages used to initiate the competition and after the outgrowth of the competition.

### Endosialidase status of phage isolates

In mixed competition assays, it was necessary to determine the proportion of phages carrying or lacking the endosialidase gene. We used a functional assay to determine this status. For both the initial phage mix and the final, post-competition mix, the phage suspension was plated on LB plates using IJ1133 (which does not discriminate against either phage type). Isolated plaques were transferred by sterile toothpick to a lawn of 98% EV36 (grown 24hr in M9 glucose) and 2% IJ1133 (grown 12-24 hr in LB). After 12-24 hr, plaques formed around the stabs exhibited a clear, broad halo if and only if the phage carried endosialidase.

### Statistics

Many assays measured amplification. The basic model for amplification is that the final phage count (*P*_*F*_) equals the initial phage count (*P*_*I*_) times the amplification factor (*A*):

(3) $$P_{F}=A\cdot P_{I}\;,$$

or equivalently

(4) $$\text{log}(P_{F})-\text{log}(P_{I})=A.$$

We wish to test the null model that amplification of one phage type (*P*) equals that of another (π),

(5) $$\text{log}(P_{F})-\text{log}(P_{I})=A=\text{log}(\pi_{F})-\text{log}(\pi_{I}).$$

When the initial phage count can be determined for each individual amplification, then

(6) $$\text{log}(P_{F,i})-\text{log}(P_{I,i})$$

can be reduced to a single value for each assay plate (*i*), and a standard t-test be performed on the values obtained from multiple plates assayed for each phage type. However, in some cases the initial counts cannot be known because plaques are not evident, and it becomes necessary to assay the initial counts independently of the final counts. In this case, we can use the model

(7) $$\bar{\text{log}(P_{F})}-\bar{\text{log}(P_{I})}=\bar{\text{log}(\pi_{F})}-\bar{\text{log}(\pi_{I})},$$

where the bar $\left(\bar{\left(\right)}\right)$ represents the mean of (). For this test, independent measures of the initial phage counts can be performed on a sensitive host and the difference of the four means subjected to a t-test with the appropriately adjusted degrees of freedom [26].

## Competing interests

The authors declare no competing interests.

## Author’s contributions

Nearly all lab work was done by EGG, and many of the protocols were developed by him. The basic design of the study and preliminary lab work was done by JJB, who also wrote the paper, did the modeling and the statistics. IJM helped resolve early ambiguities about growth of T7 on EV36 in broth and provided advice about cloning methods with the T7 vector and endosialidase; he also suggested and provided the C74 phage and the host strains. All authors read and approved the final manuscript.
